# Diagnosing mitochondrial, neurogastrointestinal leukoencephalopathy requires mutations in *TYMP1*, *POLG1*, *LIG1*, or *RRM2B*

**DOI:** 10.1186/s13256-023-03916-y

**Published:** 2023-04-14

**Authors:** Josef Finsterer

**Affiliations:** Neurology and Neurophysiology Center, Postfach 20, 1180 Vienna, Austria

**Keywords:** MNGIE, Mitochondrial disorder, Vomiting, White matter lesions, TYMP1, Lactic acidosis

**Letter to the Editor**

With interest we read the article by Jiang *et al.* about a 23-year-old female with mitochondrial neurogastrointestinal encephalopathy (MNGIE) [1]. The diagnosis MNGIE was established solely on the basis of the clinical presentation without documentation of a causative mutation [1]. Clinical manifestations of the obviously mitochondrial disorder (MID) included nonradiating, postprandial epigastric pain, bilious emesis, weight loss for 3 months, and lower limb weakness for 3 weeks [1]. Workup revealed dissociated sensory disturbances, ophthalmoparesis, gastrointestinal reflux, gastroparesis, demyelinating, sensorimotor neuropathy, myopathy, and extensive leukoencephalopathy [1]. The study is compelling but raises concerns that require further discussion.

The main limitation of the study is that the diagnosis “MNGIE” was established without documentation of a causative mutation [1]. There was also no measurement of pyridine or dihydropyridine in the urine. MNGIE is usually associated with pyrimidinuria and dihydropyrimidinuria. There was no measurement of thymidine phosphorylase enzyme activity, which is usually reduced in MNGIE. There was also no measurement of plasma concentrations of thymidine and deoxyuridine, which are usually increased in MNGIE.

Another limitation is that no family history was provided. We should be told if any first-degree relative was clinically affected. Missing are also cerebrospinal fluid (CSF) investigations to assess if CSF lactate was elevated; magnetic resonance spectroscopy (MRS) to test for cerebral lactic acidosis; and muscle biopsy to confirm myopathy as suggested upon electromyography.

We disagree with the statement in the abstract that MNGIE is only due to variants in *TYMP1* [1]. MNGIE has been also reported in patients carrying mutations in *POLG1*, *LIG1*, or *RRM2B* [2–4].

We also disagree with the statement in the abstract that leukoencephalopathy in MNGIE is asymptomatic [1]. On the contrary, patients with MNGIE commonly manifest with mental retardation, seizures, or features of autism [5].

The patient had ophthalmoparesis, but double vision was not reported [1]. What is the explanation for the patient not complaining about double vision?

The daily dosage of coenzyme-Q (CoQ10) was only 50 mg [1]. It should be mentioned why CoQ10 was given at such a low dosage. The usual daily dosage of CoQ10 is 300 mg per day.

Overall, the study carries obvious limitations that require reevaluation and discussion. Clarifying these weaknesses would strengthen the conclusions and could improve the study. Diagnosing MNGIE requires documentation of a causative mutation in *TYMP1*, *POLG1*, *LIG1*, or *RRM2B*. Arguments against MNGIE in the index patient are that there was no ptosis and no clinical cerebral abnormality.

## Data Availability

All data are available from the corresponding author.
